# Supplementary material for: Evaluating the Arrhenius equation for developmental processes
Source: Mol Syst Biol. 2021 Aug 20;17(8):e9895. doi: 10.15252/msb.20209895 (PMC8377445; doi:10.15252/msb.20209895)
Supplement: Supplementary file 2 — Appendix [file MSB-17-e9895-s003.pdf]

Appendix Table of Contents:

1) Appendix Figures
   a) Appendix Figure S1: Process for collecting developmental time-series at various temperatures.
   b) Appendix Figure S2: Log transformed developmental time-series data.
   c) Appendix Figure S3: Apparent activation energies spaced by developmental time and distribution of bootstrapped apparent activation energies.
   d) Appendix Figure S4: ANCOVA to compare activation energies and BIC to compare quadratic versus linear fit over the viable temperature range.
   e) Appendix Figure S5: BIC analysis of simulated 1000x coupled reaction networks with added experimental noise through Monte Carlo simulations explore network's ability to generate non-linear behavior to contribute to nonlinearity.
   f) Appendix Figure S6: Comparison of enzymatic reaction rates at different substrate concentrations demonstrates zero-order kinetics.
2) Mathematical Derivations
   a) Multi-reaction Network Math
   b) Concavity Math

Appendix Figures

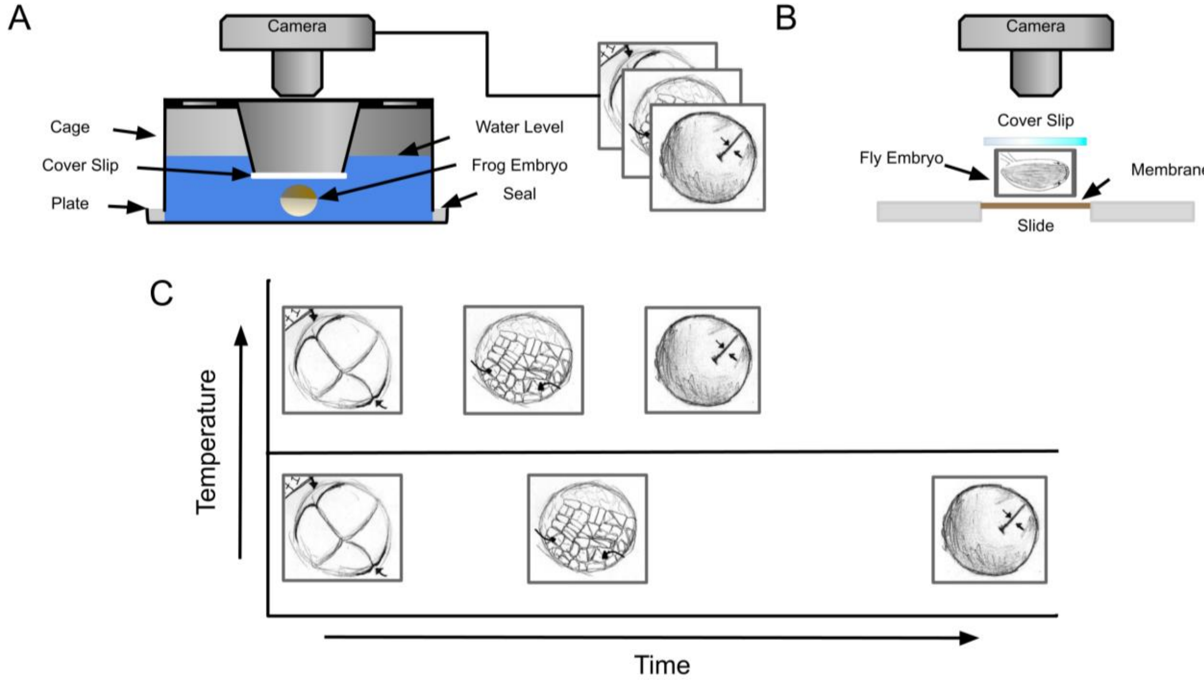

Appendix Figure S1: Process for collecting developmental time-series at various temperatures. A) A schematic of the process used to collect frog developmental data in a 3D printed aquatic cage. The cage ensures that the embryos stay in focus despite water level changes due to evaporation. Images were continuously acquired every 0.5 to 1 minute. The entire setup was held in a temperature-controlled chamber. **B)** Shown here is the process for setting up fly embryos in Halocarbon oil on an air permeable slide for time-lapse imaging. Images were acquired in a temperature-controlled room. **C)** Embryos were recorded at various temperatures. Various milestones were scored and graphed against their temperature.

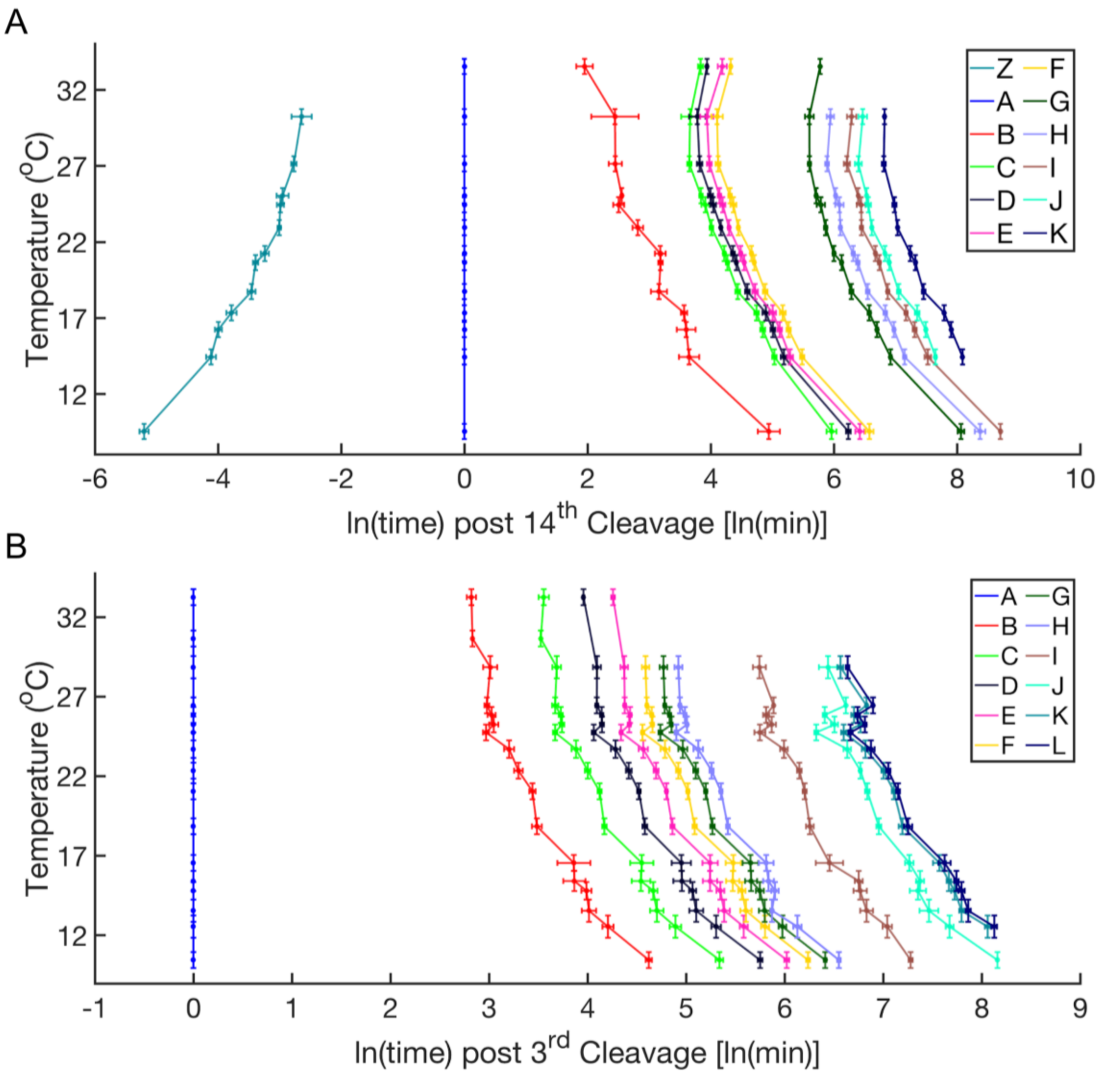

Appendix Figure S2: Log transformed developmental time-series data. A) As Figure 1C but plotting log transformed time (n = 2 -13 biological replicates per temperature). Error bars in time indicate standard deviation among replicates. Error bars in temperature represent the standard error (+/- 0.5 ^{o}C) of the thermometer used when recording temperature. **B)** As Figure 1D but plotting log transformed data. (n = 1 - 23 biological replicates per temperature).

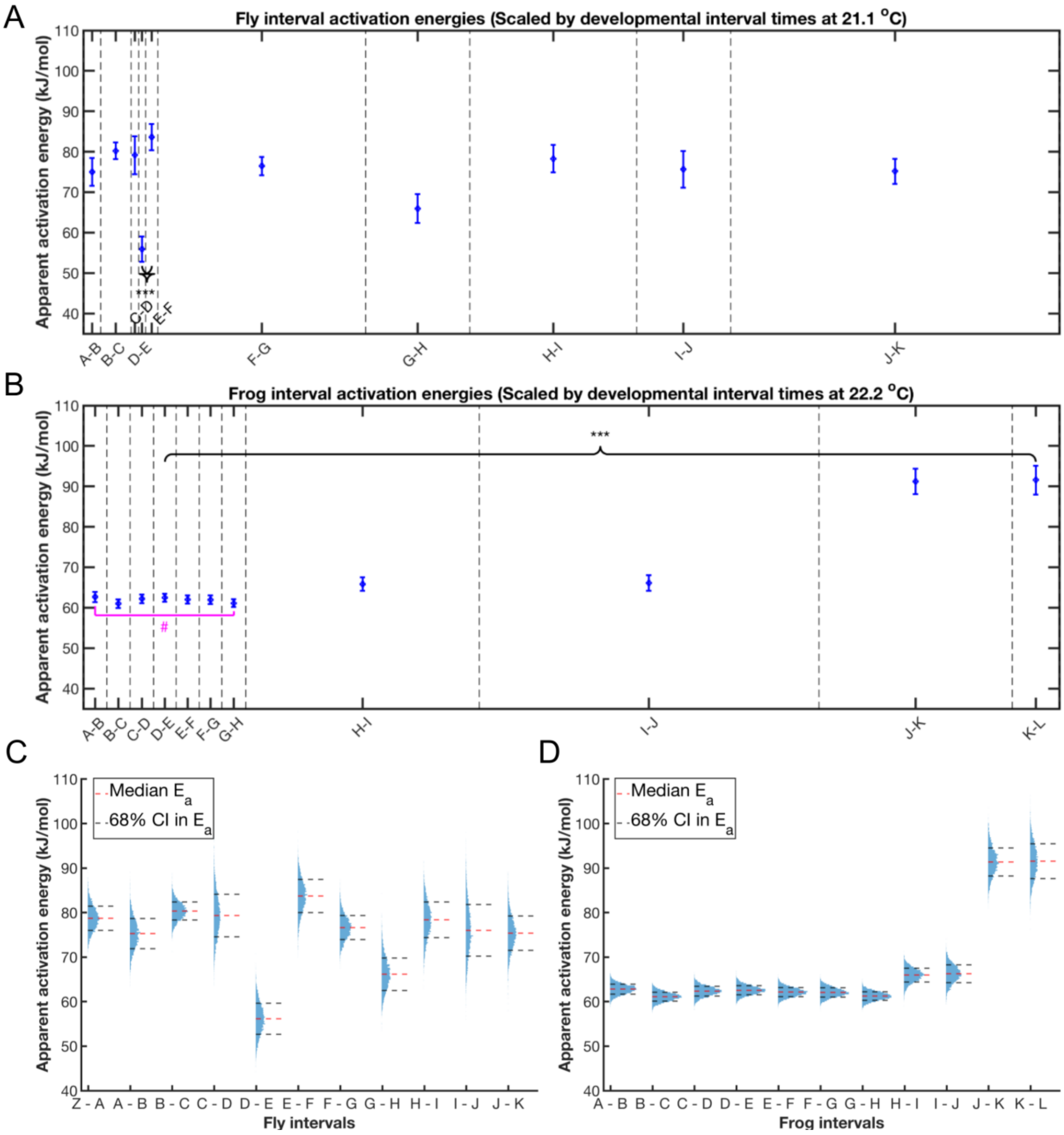

Appendix Figure S3: Apparent activation energies spaced by developmental time and distribution of bootstrapped apparent activation energies. A) As Fig. 2C, but the x-axis has been scaled by mean developmental timings of each stage at 21.1 ^{o}C. Grey vertical lines mark the borders of the developmental intervals as a portion of development from Z - K. **B)** As Fig. 2D, but the x-axis has been scaled by mean developmental timings of each stage at 22.2 ^{o}C. Grey vertical lines mark the borders of the developmental intervals as a portion of development from A - L. **C)** Histograms of bootstrapped apparent activation energies in fly calculated from 5000x bootstrapped fits on data used to generate Fig. 2C. Displayed is the median (dashed red line) as well as 68% confidence intervals (dashed black line). **D)** As (C) but bootstrapping was performed on frog data used to generate Fig. 2D.

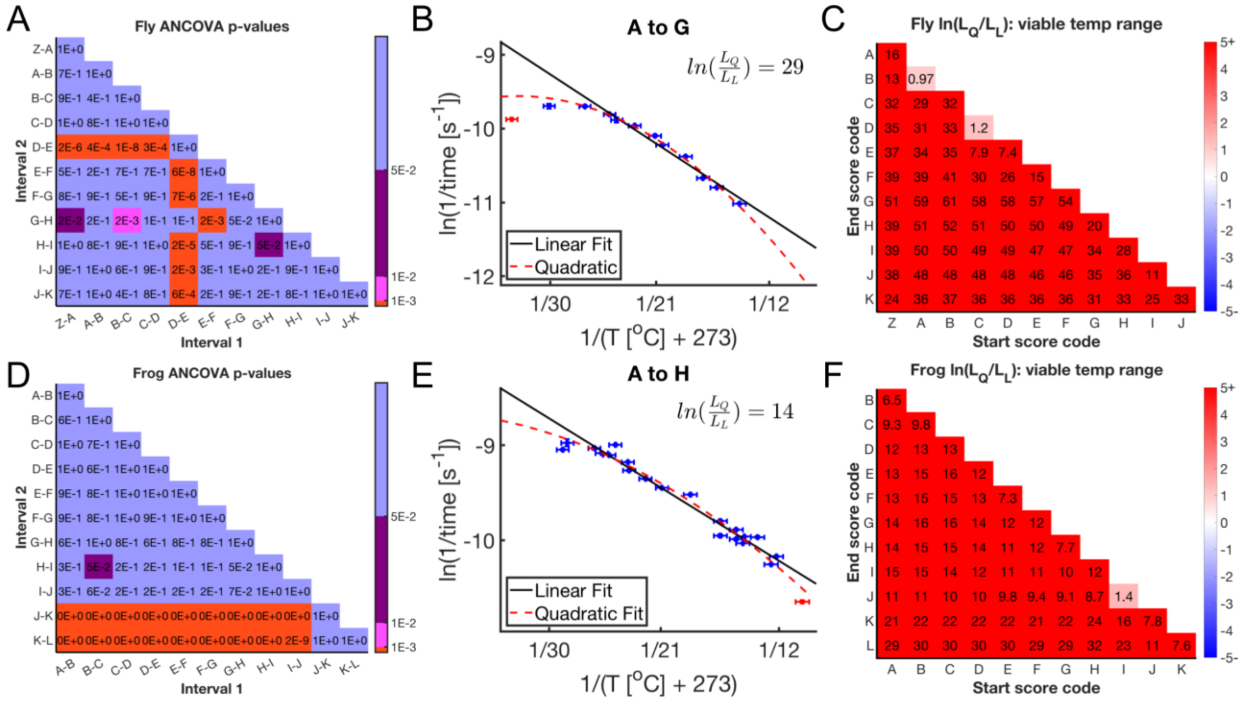

Appendix Figure S4: ANCOVA to compare activation energies and BIC to compare quadratic versus linear fit over the viable temperature range. A) p-values between fly developmental intervals using ANCOVA calculated to determine the probability of observing difference between activation energies for every combination of fly developmental intervals shown in Fig. EV2C. Blue marks p-values above 5E-2, purple marks ≤5E-2, pink marks ≤1E-2, and red marks ≤1E-3. **B)** Linear (solid black) and quadratic (dashed red) fits calculated for an example fly developmental intervals from 14th Cleavage to Beginning of Germband Retraction over the viable temperature range where embryos survive until First Breath (blue data, excluding red data). BIC was used to test model preference for a quadratic fit, reported in the top right as the log ratio likelihood for quadratic over linear for this temperature range. **C)** Shown is a heatmap reporting the natural log ratio likelihood of quadratic over linear fit preference for all developmental intervals in fly development over the viable temperature range. Intervals are marked with their beginning event on the X-axis, and their ending event on the Y-axis. Red represents a preference for quadratic while blue represents a preference for a linear fit. **D)** As (A) but for calculating frog p-values between slopes of developmental intervals shown in Fig. EV2D. **E)** As (B) but for frog 3rd to 10th cleavage over the viable temperature range where embryos survive until Late Neurulation. **F)** As (C) but for all possible frog intervals.

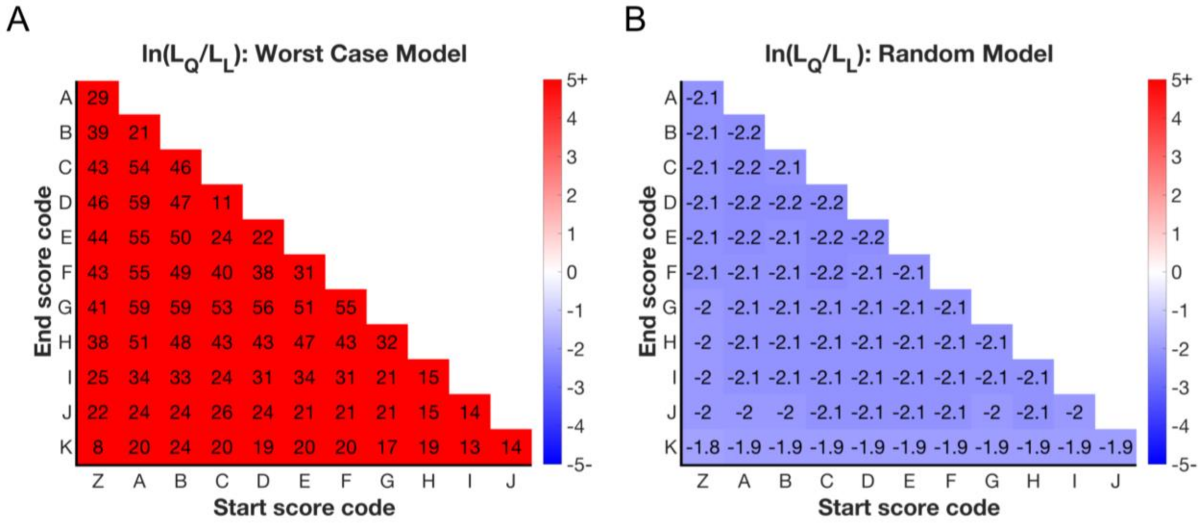

Appendix Figure S5: BIC analysis of simulated 1000x coupled reaction networks with added experimental noise through Monte Carlo simulations explore network's ability to generate non-linear behavior to contribute to nonlinearity. A) Heatmap of the natural log ratio of penalized likelihoods (quadratic/linear) comparing fit preference for simulated fly interval data, based on measured data-points, generated from equation (3) with 1000 parameters optimized for maximum curvature at 295 ^{o}K. Standard errors in ln(k) were extracted from our biological experiments for all developmental fly interval at every temperature and used to introduce noise into simulated data's ln(k). Results shown for each interval are the mean of 100 such simulations, where each replicate perturbed simulated embryos' ln(k) values by sampling noise from a normal distribution using the corresponding fly interval's standard error at each respective temperature. Noise in temperature was sampled from a normal distribution using the standard error of our thermometer. **B)** As (A) but using a 1000x coupled reaction network of randomly selected E_a and A.

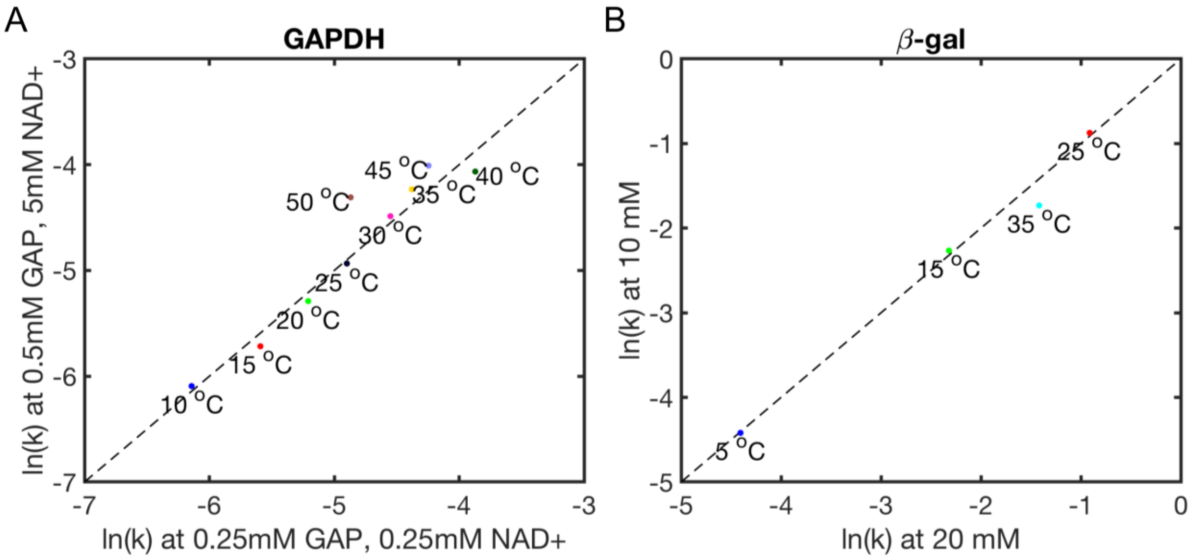

Appendix Figure S6: Comparison of enzymatic reaction rates at different substrate concentrations demonstrates zero-order kinetics. A) Activity for GAPDH assay with 0.5 mM GAP/5 mM NAD+ (used for Fig. 4D) is similar to activity with 0.25 mM GAP/0.25 mM NAD+. **B)** Activity of β-galactosidase for 10 mM (Fig. EV 5) is similar to activity with 20 mM ONPG.

Mathematical derivations

Multi-reaction Network

The following is a derivation for an equation modeling the temperature dependence of a sequential multi-reaction system of reactions, each of which individually follow Arrhenius.

First, we show that a relaxation time scale formulation yields the known $\tau = \frac{1}{k}$, where τ is the reaction network's time constant and k is the rate constant, assuming a simple transition from some stage $A \rightarrow B$. Here, A_0 is the initial amount of A and $B(t)$ is the amount of B at time t, and we define a function $R(t)$, which defines the fraction of the total mass that hasn't been converted to the final product.

$$R_{A\rightarrow B}(t) = \frac{A_0 - B(t)}{A_0}$$

If we take the difference between two points on $R(t)$ we get the incremental change:

$$R_{A\rightarrow B}(t) - R(t+dt) = -\frac{\partial R}{\partial t}dt$$

Now we can define the network's time constant, τ:

$$\tau = \int_0^\infty t\left(-\frac{\partial R}{\partial t}\right)dt$$

Integrating we arrive at the following expressions:

$$\tau = tR(t)|_0^\infty + \int_0^\infty R(t)\,dt$$

$$\tau = \int_0^\infty R(t)\,dt$$

Before fully solving this integral we must first simplify $R(t)$, where $R(t) = \frac{A_0 - B(t)}{A_0}$. Here we can substitute $A_0 - A_0e^{-kt}$ for $B(t)$, giving:

$$R(t) = \frac{A_0 - (A_0 - A_0e^{-kt})}{A_0}$$

And finally:

$$R(t) = e^{-kt}$$

Now we substitute e^{-kt} for $R(t)$ in $\tau = \int_0^\infty R(t)dt$, and solve for τ

$$\tau = \int_0^\infty e^{-kt}dt$$

$$= \frac{1}{k}e^{-kt}|_0^\infty$$

$$\tau = \frac{1}{k}$$

Therefore, for a simple transition from some stage $A \rightarrow B$ a relaxation formulism captures the known relationship of $\tau = \frac{1}{k}$, and by substituting in the Arrhenius equation, $\tau = \frac{e^{Ea/RT}}{A}$.

Now we expand this approach to a more complicated transition series, from $A \rightarrow B \rightarrow C$ to determine if a relaxation formulism can be generalizable to arbitrarily high reaction transitions or stages. Let k_1 and k_2 be the rate constants for the first and second steps of the reaction. Then, we start by defining our relaxation function $R(t)$:

$$R(t) = \frac{A_0 - C(t)}{A_0}$$

The kinetics of the reactants over time $A(t)$, $B(t)$, $C(t)$ can be defined as follows:

$$A(t) = A_0 e^{-k_1 t}$$

$$B(t) = A_0 \frac{k_1}{k_2 - k_1}(e^{-k_1 t} - e^{-k_2 t})$$

$$C(t) = A_0 \frac{1}{k_2 - k_1}\left(k_2(1 - e^{-k_1 t}) - k_1(1 - e^{-k_2 t})\right)$$

Substituting these values into our relaxation function $R(t)$ and simplifying gives:

$$R(t) = \frac{A_0 - A_0 \frac{1}{k_2 - k_1}\left(k_2(1 - e^{-k_1 t}) - k_1(1 - e^{-k_2 t})\right)}{A_0}$$

$$= 1 - \frac{1}{k_2 - k_1}\left(k_2(1 - e^{-k_1 t}) - k_1(1 - e^{-k_2 t})\right)$$

$$= \frac{(k_2 - k_1) - (k_2 - k_2 e^{-k_1 t} - k_1 + k_1 e^{-k_2 t})}{k_2 - k_1}$$

$$= \frac{k_2 - k_1 - k_2 + k_2 e^{-k_1 t} + k_1 - k_1 e^{-k_2 t}}{k_2 - k_1}$$

$$R(t) = \frac{k_2 e^{-k_1 t} - k_1 e^{-k_2 t}}{k_2 - k_1}$$

We can now substitute $R(t)$ back into our definition for a network's time constant $\tau = \int_0^\infty R(t)dt$, giving the following:

$$\tau = \frac{1}{k_2 - k_1} \int_0^\infty (k_2 e^{-k_1 t} - k_1 e^{-k_2 t}) dt$$

Solving the integral and simplifying this equation gives the following:

$$\tau = \frac{1}{k_2 - k_1} \left(k_2 \int_0^\infty e^{-k_1 t}\, dt - k_1 \int_0^\infty e^{-k_2 t}\, dt \right)$$

$$= \frac{1}{k_2 - k_1} \left(k_2 \left(\frac{1}{k_1} e^{-k_1 t} |_0^\infty \right) - k_1 \left(\frac{1}{k_2} e^{-k_2 t} |_0^\infty \right) \right)$$

$$= \frac{\frac{k_2}{k_1} - \frac{k_1}{k_2}}{k_2 - k_1}$$

$$= \frac{\frac{k_2^2 - k_1^2}{k_1 k_2}}{k_2 - k_1}$$

$$= \frac{(k_2 - k_1)(k_2 + k_1)}{(k_1 k_2)(k_2 - k_1)}$$

$$= \frac{k_2 + k_1}{k_1 k_2}$$

$$\tau = \frac{1}{k_2} + \frac{1}{k_1}$$

We can now substitute in the Arrhenius equation, $k = Ae^{-E_a/RT}$, for k to give us τ in terms of Arrhenius parameters A and E_a.

$$\tau(T) = \frac{e^{E_{a_2}/RT}}{A_2} + \frac{e^{E_{a_1}/RT}}{A_1}$$

Here we see a deterministic pattern, and further analysis (not shown) of higher order reactions series confirms its generality. The above equation can be simplified as a summation:

$$\tau(T) = \sum_{i=1}^{n} \frac{e^{E_{a_i}/RT}}{A_i} \qquad (2)$$

Since $\tau = \frac{1}{k}$ still holds true we can put equation (2) in terms of k instead of τ and arrive at a form close to the linearized Arrhenius equation, $ln(k) = ln(A) - \frac{E_a}{RT}$.

$$k(T) = \tau(T)^{-1}$$

Substitute $\sum_{i=1}^{n} \frac{e^{E_{a_i}/RT}}{A_i}$ in for $\tau(T)$

$$k(T) = \left(\sum_{i=1}^{n} \frac{e^{E_{a_i}/RT}}{A_i}\right)^{-1}$$

$$ln(k) = ln\left(\left(\sum_{i=1}^{n} \frac{e^{E_{a_i}/RT}}{A_i}\right)^{-1}\right)$$

Simplify exponents:

$$ln(k) = -ln\left(\sum_{i=1}^{n} \frac{e^{E_{a_i}/RT}}{A_i}\right)$$

Therefore, for a sequential series of n reactions, each *i^th^ reaction* of which strictly follows Arrhenius, then the Arrhenius plot for such a system should follow the below equation.

$$ln(k) = -ln\left(\sum_{i=1}^{n} \frac{e^{E_{a_i}/RT}}{A_i}\right) \qquad (3)$$

Concavity

The following is a derivation for the concavity of equation (3), which models the temperature dependence of a sequential multi-reaction system of reactions, each of which individually follows Arrhenius.

We begin with equation (3): $\ln(k) = -\ln\left(\sum_i \frac{e^{E_{a_i}/RT}}{A_i}\right)$. To simplify calculations, let $x_i = \frac{e^{E_{a_i}/RT}}{A_i}$, and let $c_i = E_{a_i}/R$. Then, $\frac{dx_i}{d(1/T)} = c_i x_i$.

Now, if we let $S = \sum_i \frac{e^{E_{a_i}/RT}}{A_i} = \sum_i x_i$, then $S' = \frac{dS}{d(1/T)} = \sum_i c_i\, x_i$ and $S'' = \frac{dS}{d(1/T)} = \sum_i c_i^2\, x_i$.

Thus, we have

$$\frac{d(\ln(k))}{d(1/T)} = \frac{d}{d(1/T)}(-\ln(S)) = -\frac{S'}{S}\ ,$$

and then

$$\frac{d^2(\ln(k))}{d(1/T)^2} = -\frac{SS'' - (S')^2}{S^2}.$$

We claim that this second derivative is always negative. Since the denominator is a square and thus positive, it is sufficient to show that $SS'' - (S')^2 > 0$. But we know that

$$SS'' - (S')^2 = \left(\sum_i x_i\right)\left(\sum_i c_i^2\, x_i\right) - \left(\sum_i c_i\, x_i\right)^2.$$

Now, consider the coefficient of $x_i x_j$ in this difference. If $i = j$, then both terms will give a coefficient of c_i^2, and these will cancel each other out in the difference. Otherwise, if $i \neq j$, then the first term will give a coefficient of $(c_i^2 + c_j^2)$, while the second term will give a coefficient of $2c_i c_j$. Therefore, the resulting term in the difference of these products will be

$$(c_i^2 + c_j^2 - 2c_i c_j)x_i x_j = (c_i - c_j)^2 x_i x_j.$$

Since each x_i is positive (being the quotient of an exponential, which is always positive, and a positive prefactor A_i), and since $(c_i - c_j)^2$ is a square, the entire term $(c_i - c_j)^2 x_i x_j$ is positive (unless $c_i = c_j$, in which case it's equal to 0). Therefore, unless all the c_i's, and thus all the E_{a_i}'s, are equal, we have that $SS'' - (S')^2 > 0$, and so the second derivative $\frac{d^2(\ln(k))}{d(1/T)^2}$ is always negative. This means that the graph of $\ln(k)$ versus $1/T$ is always concave down.
