# Supplementary material for: Evaluating the Arrhenius equation for developmental processes
Source: Mol Syst Biol. 2021 Aug 20;17(8):e9895. doi: 10.15252/msb.20209895 (PMC8377445; doi:10.15252/msb.20209895)
Supplement: Supplementary file 3 — Expanded View Figures PDF [file MSB-17-e9895-s001.pdf]

Expanded View Figures

Figure EV1. Scored developmental events and coefficient of variation (CV) analysis.

A Sketches of 12 developmental scores determined to be the most reproducible, in *D. melanogaster*. Please see Materials and Methods and Movie EV1 for definition of scoring criteria.

B Seven additional developmental events in fly embryos that we did not pursue due to poor reproducibility i.e. cut score. The number score code is used in the following CV analysis.

C We calculated CVs using preliminary data for every developmental time interval between the 19 scores (described in Materials and Methods) we considered investigating (Dataset EV1). CVs are calculated for each of the 6 different temperatures (n = 3–5 biological replicates per temperature), and the mean CV is then displayed for each interval. CV's are displayed as percentages. The 11 most reproducible intervals for neighboring scores (diagonal) are shown in green. Intervals shown in red had their associated score (numbers) cut from our investigation.

D Sketches of 12 developmental event we investigated in *X. laevis* determined most reproducible. Please see Materials and Methods and Movie EV2 for definition of scoring criteria.

E As (B) but for four additional frog developmental events not included in our final analysis.

F As (C) but for intervals calculated from early data, between 16 frog developmental scores considered (described in Material and Methods) averaged over 16 temperatures (n = 2–6 biological replicates per temperature) (Dataset EV2). The 11 most reproducible intervals for neighboring scores (diagonal) are shown in green.

© 2021 The Authors

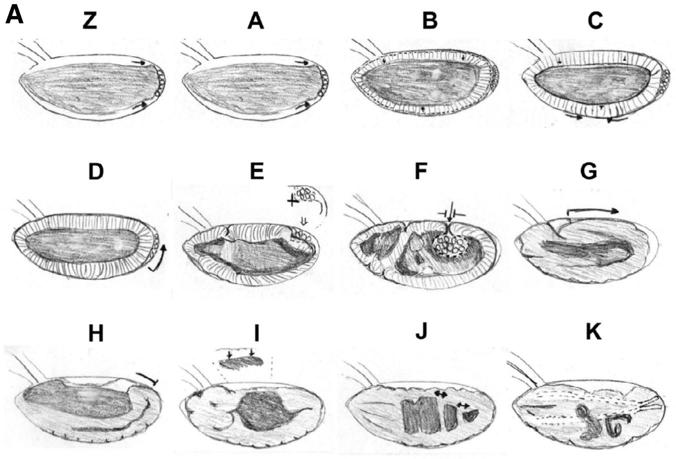

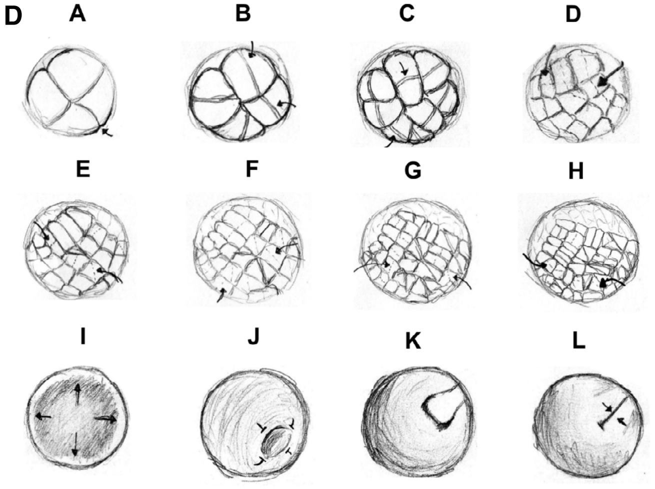

B

Cut score name	Cut score code
Beginning of Posterior MidGut Opening	1
Head Invagination	2
Gut Movement	3
Head Movement	4
First Breath Ends	5
Gut Contents Migration	6
Hatching	7

E

Cut score name	Cut score code
Post Neurulation	1
De-Sphericalization	2
Contraction of Hatching Gland	3
First Anterior Twitch	4

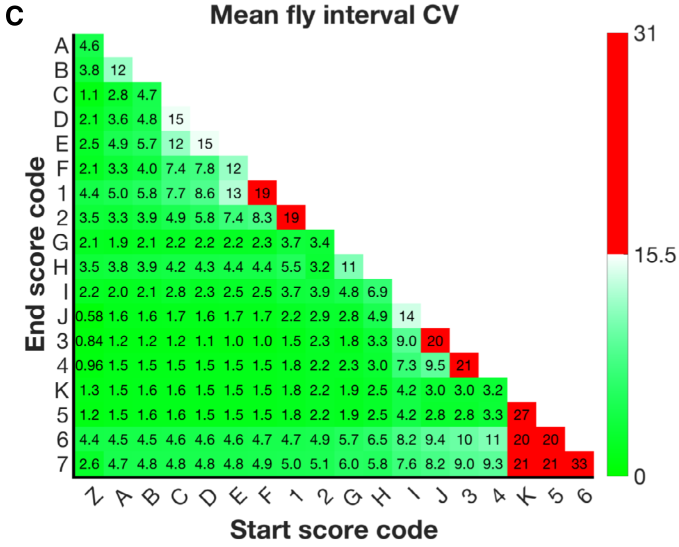

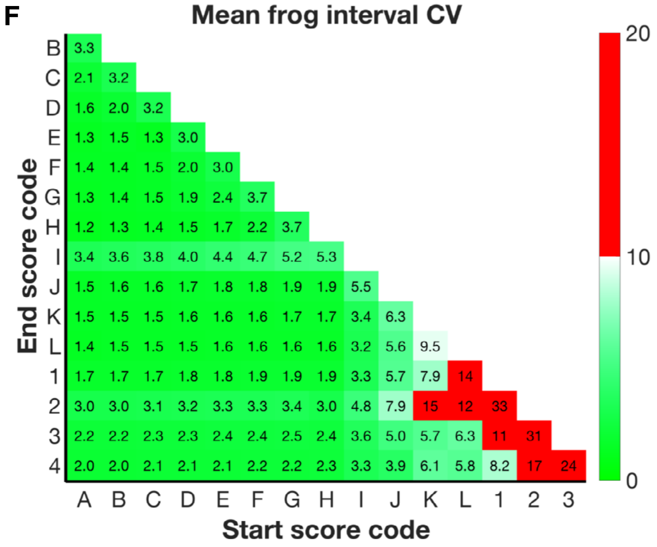

Figure EV1.

©2021 The Authors

Figure EV2. Arrhenius plots for fly and frog.

A Shown are Arrhenius plots (similar to Fig 2A) for developmental intervals between 12 adjacent fly developmental scores. Linear fits (solid black line) were calculated from 14.3 to 27°C. The apparent activation energy for each interval is displayed top right of each subplot. Blue data points represent temperatures viable until First Breath. Extreme temperatures that do not survive until our final scores are shown in red. A quadratic (dashed red line) is fit through all the data (red and blue). Error bars in temperature represent the standard error (± 0.5°C) of the thermometer used when recording temperature. Error bars in ln(rate) represent standard error (n = 2–13 biological replicates per temperature).

B As (A) but using 12 adjacent frog developmental scores. Linear fits are calculated from 12.2 to 25.7°C, used in Fig 2B, (n = 1–23 biological replicates per temperature). Blue data points represent temperatures viable until Late Neurulation.

© 2021 The Authors

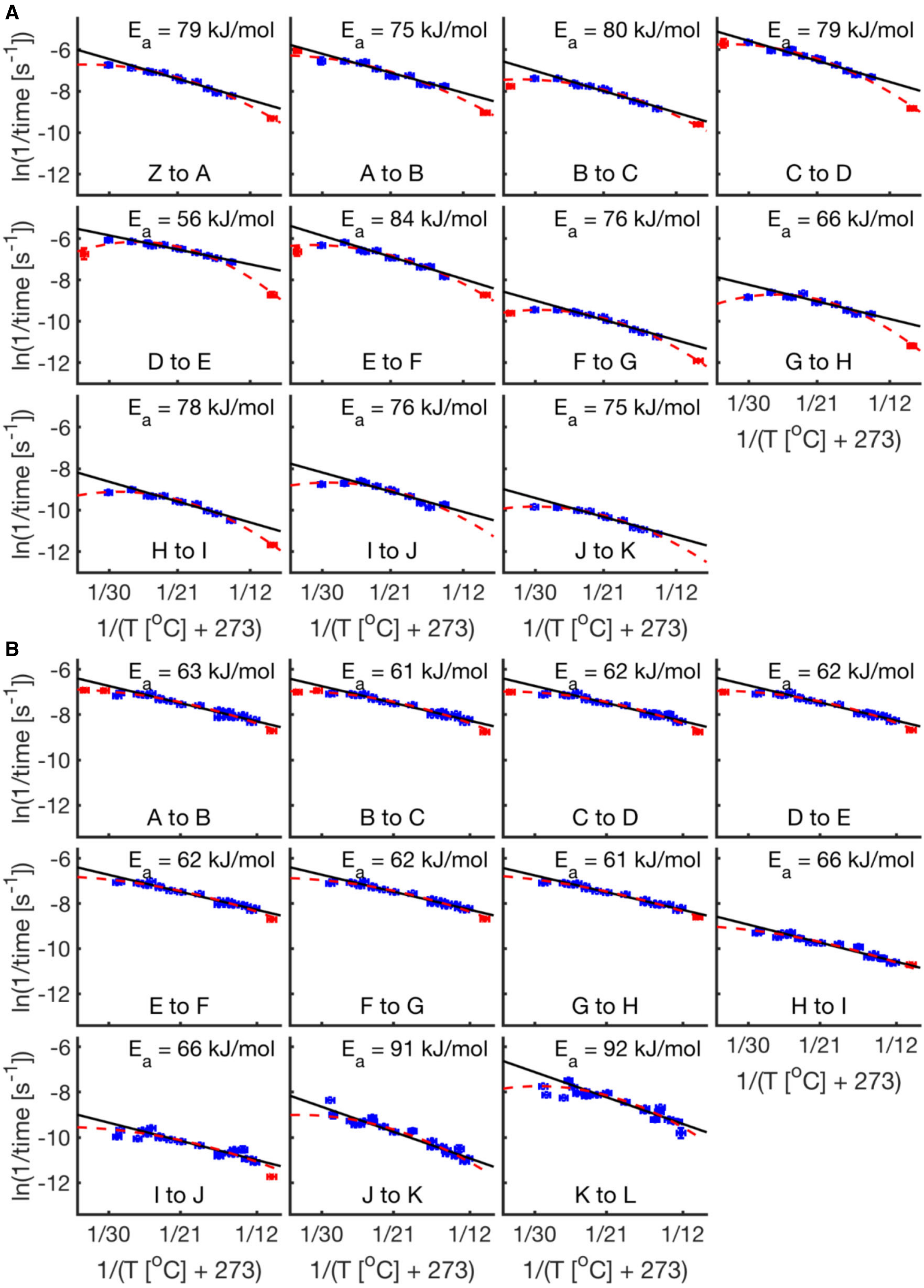

Figure EV2.

©2021 The Authors

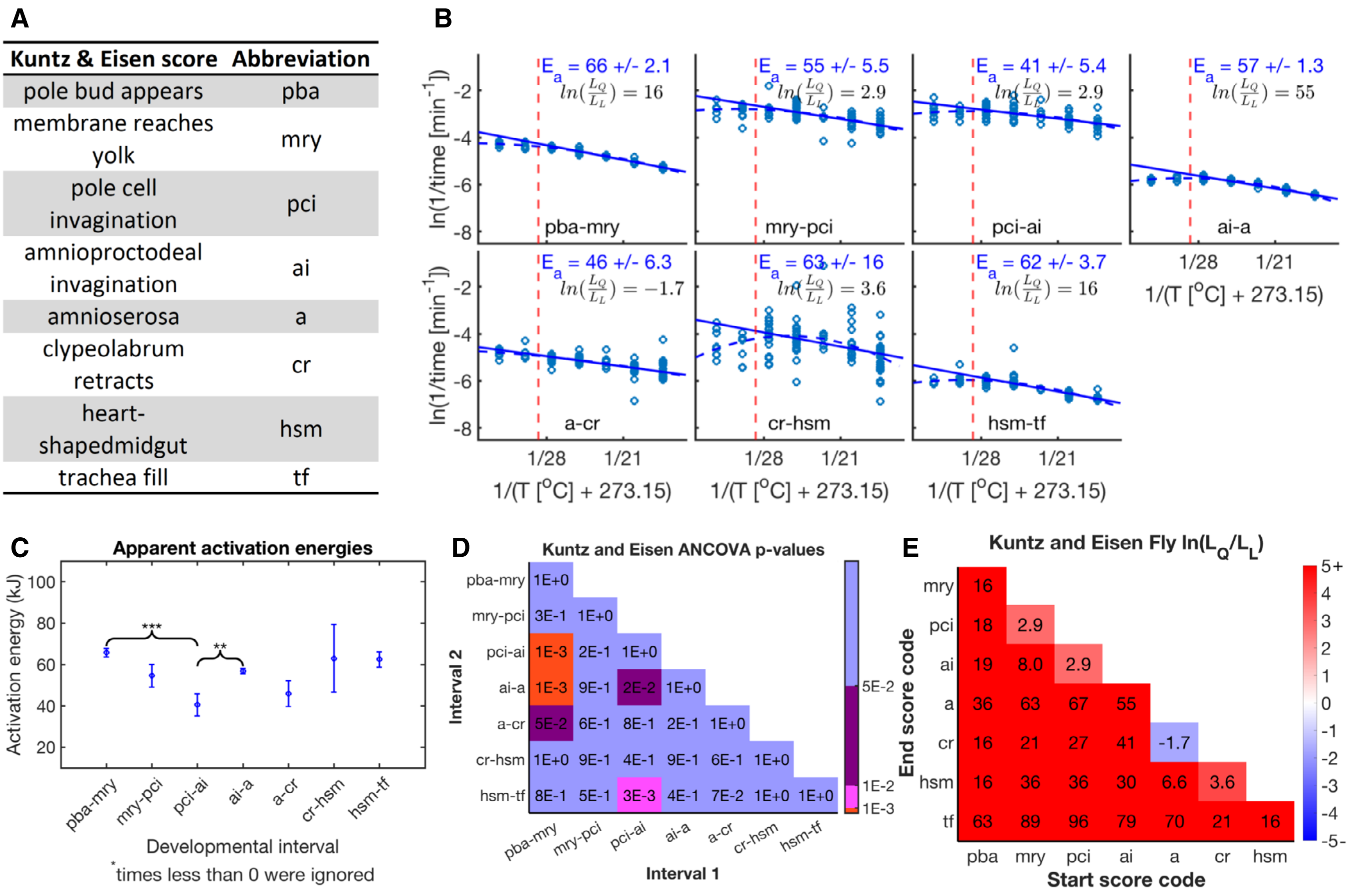

Figure EV3. Reanalysis of temperature dependence data in *Drosophila melanogaster* development from Kuntz and Eisen.

A Scores Kuntz and Eisen used and abbreviation for the remainder of this figure.

B Arrhenius plots for durations between adjacent developmental events of Kuntz and Eisen's data. We fit data for temperatures of 27.5°C and below (dashed red line, i.e., core temperature range) via linear regression (solid blue line). Shown is the apparent activation energy plus/minus the 68% confidence interval. Additionally, we fit the entire temperature range with quadratic (dashed blue line) and linear fits. BIC was also calculated and is shown here as the natural log ratio likelihood for quadratic over linear fit and displayed in black.

C Apparent activation energies over the core temperature regime are shown. Error bars show the 68% confidence intervals. Black braces point out example developmental intervals that have significantly different apparent activation energies. '**'*P*-value < 0.01, '***'*P*-value < 0.001.

D Shown are *P*-values between all developmental intervals. Blue marks *P*-values above 5E-2, purple marks ≤ 5E-2, pink marks ≤ 1E-2, and red marks ≤ 1E-3.

E Shown are natural log ratio of likelihoods for quadratic over linear fits for all possible developmental intervals, marked by their starting and ending scores, using Kuntz and Eisen's data over all temperatures. Blue signifies a preference for linearity; red signifies a preference for quadratic behavior.

A

Stage	Fly lnA	Fly Ea
Z to A	25	79
A to B	24	75
B to C	25	80
C to D	26	79
D to E	16	56
E to F	27	84
F to G	21	76
G to H	18	66
H to I	22	78
I to J	22	76
J to K	20	75

B

Stage	Frog lnA	Frog Ea
A to B	18	63
B to C	17	61
C to D	18	62
D to E	18	62
E to F	18	62
F to G	18	62
G to H	17	61
H to I	17	66
I to J	17	66
J to K	28	91
K to L	29	92

C

$$\ln(\kappa) = -\ln\left(\sum_{i=1}^{n} \frac{e^{\frac{E_{a_i}}{RT}}}{A_i}\right)$$

D

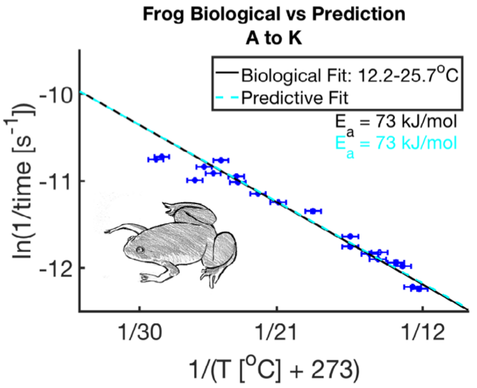

Figure EV4. Parameters for linear fits and sequential linear model prediction capture temperature dependence of entire embryonic development well.

A A table showing fly intervals marked by their start and ending scores and their empirically determined prefactors (lnA) and apparent activation energy (E_a) from Fig EV2A.

B As (A) but for frog prefactors and apparent activation energy calculated empirically from Fig EV2B.

C Our predictive equation determined in equation (3) for an assumed sequential linear network.

D Prediction of ln(k) for the composite network (dashed cyan) is overlaid on the empirical data (blue and red error bars) and linear fit (solid black) for this developmental interval (A to K). Also shown are the color-coded E_as calculated for each fit over the temperature interval 12.2–25.7°C (n = 100 independent biological measurements). Error bars in temperature represent the standard error (± 0.5°C) of the thermometer used when recording temperature. Error bars in ln(rate) represent standard error (n = 2–10 biological replicates per temperature).

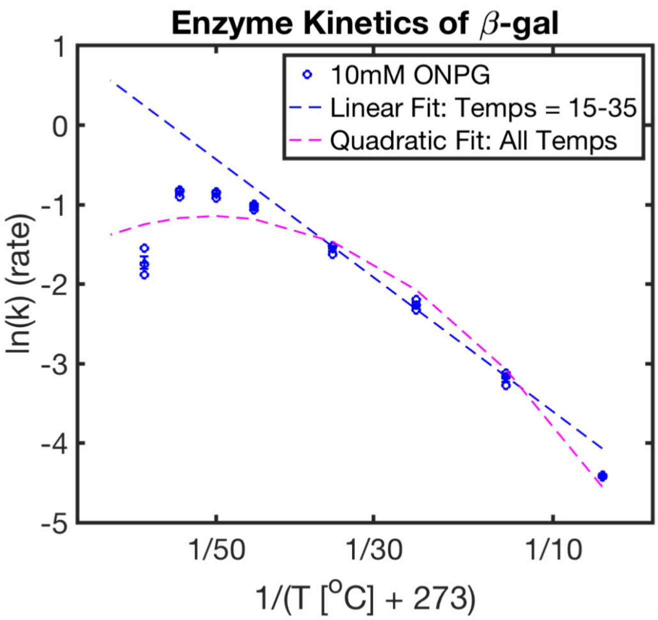

Figure EV5. Assay of β-galactosidase activity over its viable temperature range under 0-order kinetic conditions.

Plotted here is the Arrhenius plot for this conversion. Replicates (blue circles) are fit with a linear fit (dashed blue line) from 15 to 35°C and a quadratic fit (dashed magenta) over the entire viable temperature range. Standard error is shown as blue error bars (n = 3 technical replicates per temperature). Reaction was run using 0.25 U/ml β-galactosidase.

 ©2021 The Authors
