# Supplementary material for: Evaluating the Arrhenius equation for developmental processes
Source: Mol Syst Biol. 2021 Aug 20;17(8):e9895. doi: 10.15252/msb.20209895 (PMC8377445; doi:10.15252/msb.20209895)
Supplement: Supplementary file 13 — Movie EV2 [file MSB-17-e9895-s011.zip › Movie EV2 480p/Movie EV2.docx]

**Movie EV2:** **Example scoring movie for Frog.** Shown is an example of a frog embryo developmental time-lapse used throughout our analysis to extract developmental timings (23.6 ^o^C). Additionally shown are the scoring criteria used. Specifically scores are exaggerated by pausing the video for several frames.
